# Supplementary material for: Extrahepatic Malignancies Are the Leading Cause of Death in Patients with Chronic Hepatitis B without Cirrhosis: A Large Population-Based Cohort Study
Source: Cancers (Basel). 2024 Feb 7;16(4):711. doi: 10.3390/cancers16040711 (PMC10886555; doi:10.3390/cancers16040711)
Supplement: Supplementary file 1 [file cancers-16-00711-s001.zip › cancers-2845552-supplementary.pdf]

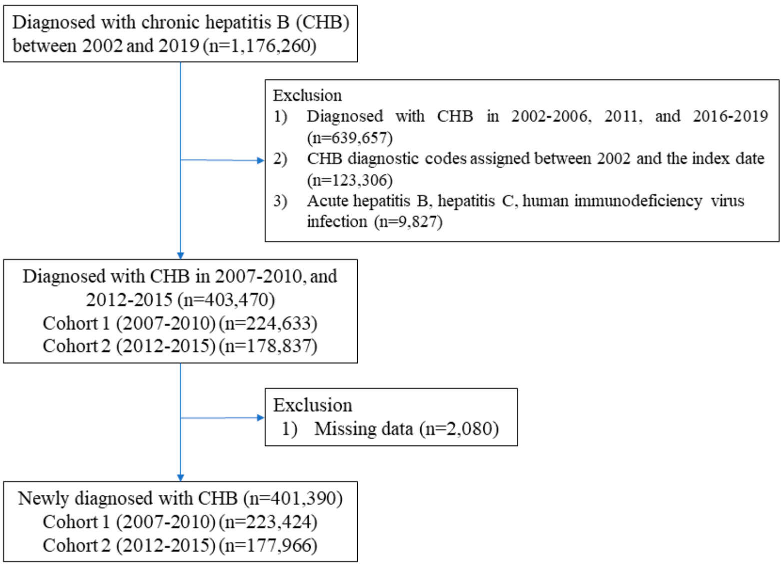

Supplementary Figure S1. Flow chart of patient selection in this study

Supplementary Table S1. Baseline characteristics of patients comparing cohort 1 *vs.* cohort 2

	Total (n=401,390)	Cohort1 (n=223,424)	Cohort2 (n=177,966)	*P*-value
Age	46.3 ± 14.7	45.0 ± 14.8	47.9 ± 14.4	<0.001
Men	220,667 (54.9)	125,025 (56.0)	95,642 (53.7)	<0.001
Body mass index, kg/m^2	23.9 ± 3.3	23.9 ± 3.2	24.0 ± 3.4	<0.001
Residence in Seoul	77,133 (19.2)	42,990 (19.2)	34,143 (19.2)	0.656
Antiviral treatment	83,380 (20.8)	58,385 (26.1)	24,995 (14.0)	<0.001
Comorbidities				
Cirrhosis	47,184 (11.8)	28,220 (12.6)	18,964 (10.7)	<0.001
Decompensated cirrhosis	10,612 (2.6)	6,601 (3.0)	4,011 (2.3)	<0.001
Hepatocellular carcinoma	40,427 (10.1)	20,458 (9.2)	19,969 (11.2)	<0.001
Hypertension	108,347 (27.0)	58,399 (26.1)	49,948 (28.1)	<0.001
Diabetes mellitus	103,296 (25.7)	54,790 (24.5)	48,506 (27.3)	<0.001
Cardiovascular disease	29,974 (7.5)	16,346 (7.3)	13,628 (7.7)	<0.001
Cerebrovascular disease	16,402 (4.1)	9,718 (4.3)	6,684 (3.8)	<0.001
Chronic kidney disease	16,929 (4.2)	9,007 (4.0)	7,922 (4.5)	<0.001
Dyslipidemia	186,245 (46.4)	88,523 (39.6)	97,722 (54.9)	<0.001
Extrahepatic malignancy	82,106 (20.5)	40,602 (18.2)	41,504 (23.3)	<0.001
Charlson Comorbidity Index score	3.7 ± 2.6	3.4 ± 2.5	4.0 ± 2.7	<0.001
Follow up duration, months	42.1 ± 42.1	49.5 ± 46.0	27.4 ± 27.9	<0.001

Variables were presented as n (%) or mean ± standard deviation.

HCC, hepatocellular carcinoma
